# Supplementary figures and images for: The effect of diluted 1% baby shampoo on biofilm reduction in chronic rhinosinusitis with nasal polyposis
Source: PeerJ. 2025 Apr 24;13:e19134. doi: 10.7717/peerj.19134 (PMC12034242; doi:10.7717/peerj.19134)

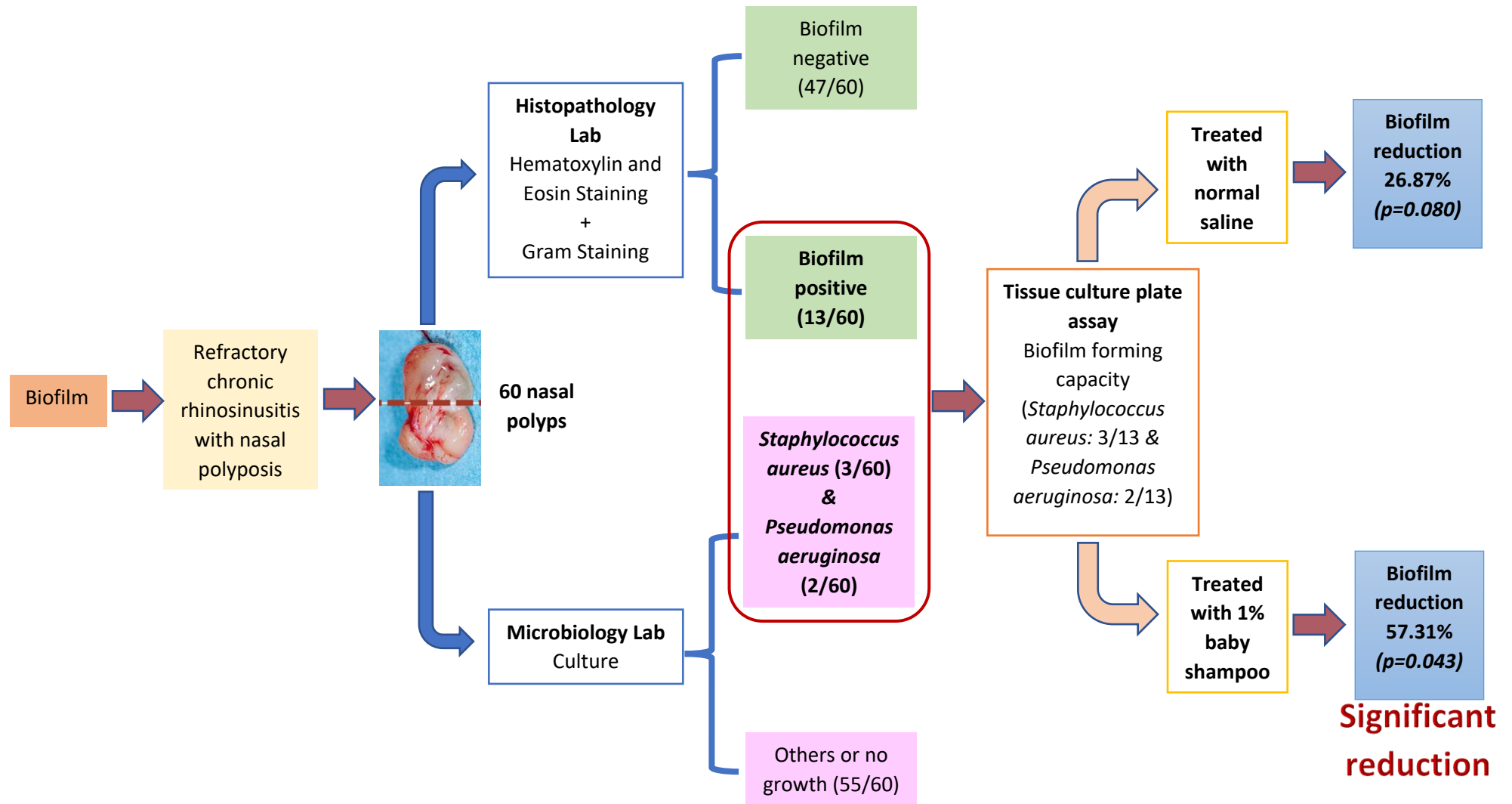

Supplement: Supplemental Information 2 [file peerj-13-19134-s002.pdf]
